# Drying of Saffron Petals as a Critical Step for the Stabilization of This Floral Residue Prior to Extraction of Bioactive Compounds

**DOI:** 10.3390/foods13233724

**Published:** 2024-11-21

**Authors:** Inmaculada Criado-Navarro, Francisco Barba-Palomeque, Pedro Pérez-Juan, Carlos A. Ledesma-Escobar, Feliciano Priego-Capote

**Affiliations:** 1 Department of Analytical Chemistry, University of Córdoba, 14071 Córdoba, Spain; q12crnai@uco.es (I.C.-N.); qa2bapaf@uco.es (F.B.-P.); q72prcaf@uco.es (F.P.-C.); 2 Chemical Institute for Energy and Environment (iQUEMA), University of Córdoba, 14071 Córdoba, Spain; 3 Maimónides Institute of Biomedical Research (IMIBIC), Reina Sofía University Hospital, University of Córdoba, 14004 Córdoba, Spain; 4 CIBER of Frailty and Healthy Ageing (CIBERFES), Carlos III Health Institute, 28029 Madrid, Spain; 5 Regulatory Council Foundation of the Protected Designation of Origin Azafrán de La Mancha, 45720 Toledo, Spain

**Keywords:** saffron, petals, drying, flavonoids, anthocyanins, evaporation

## Abstract

Saffron petals represent floral biomass generally wasted due to rapid deterioration. Previous characterization studies have revealed the presence of bioactive compounds in petals, such as flavonols and anthocyanins. Petal stabilization is a challenge for the efficient isolation of these compounds. This research evaluated three different drying techniques before the solid–liquid extraction of bioactive compounds: oven-drying (40 and 60 °C), lyophilization, and vacuum evaporation (25 and 50 °C). The characterization of the extracts allowed the annotation of 22 metabolites with a quantitative predominance of anthocyanins and derivatives of kaempferol and quercetin. Oven-drying at 60 °C was the most suitable approach for extracting minor compounds, such as crocins and safranal, at concentrations below 1 mg/g dry weight. Vacuum evaporation (50 °C) and lyophilization were the most recommended strategies for efficiently isolating flavonoids. Therefore, drying saffron petals is crucial to ensure the efficient extraction of bioactive compounds.

## 1. Introduction

Saffron is a spice obtained by dehydrating the stigmas isolated from the flower of *Crocus sativus* L. Saffron flowers are a combination of six petals, three stamens, and three red stigmas. The dried red stigmas of saffron flowers are one of the most valued spices in the world for their ability to color and flavor foods. In addition, saffron stigmas are used in cosmetics formulation and for phytotherapeutic applications [1,2]. The stigmas are the only marketable part of the plant; the rest of the plant, the floral biomass, is produced in large quantities in the production of saffron but the processing of every 78 kg of fresh flowers results in 1 kg of dried saffron [3]. Thus, saffron petals are produced in large quantities as compared to stigmas, but the former is generally not used as a food component and is usually wasted after harvesting. Saffron petals contain protein, fat, ash, fiber, and elements such as sodium, potassium, calcium, copper, iron, magnesium, zinc, and phosphorus. It also contains flavonols, carotenoids, anthocyanins, phenolic compounds, terpenoids, and alkaloids [4,5]. According to medical studies, saffron petals can be used to treat depression [6] and have antinociceptive, anti-inflammatory, and antioxidant activities [7]. The health effects of the petal extract and its antioxidant capacity suggest a possible valorization of this saffron by-product as a source of bioactive compounds that could potentially increase the economic value of this crop [8].

Drying is one of the ancient and approved methods for preserving plant material. In fact, drying is the most important step in saffron post-harvest processing, significantly affecting its physicochemical and qualitative characteristics. Drying can also create new product forms. Predicting the drying kinetics of agricultural products under different conditions is essential for process and equipment design, energy and fuel management, selection of appropriate storage, and material handling [9,10]. Temperature is one of the most influential parameters in drying since compounds such as anthocyanins are thermally sensitive. Traditionally, saffron petals were dried for four days at room temperature and two days under the sun [11]. More efficient methods are now available. Air-drying is an early process used in food preservation in which the material to be dried is exposed to a continuous flow of hot air, which evaporates the moisture content. Although it is a fast and inexpensive technique, it causes significant changes involving physical changes, chemical reactions, and biochemical effects [12]. On the other hand, vacuum evaporation is considered to be the most practical approach, offering the lowest capital cost at the highest evaporation level. In addition, it is used in industry as a concentration technique but also requires heating for a relatively long period [13]. Freeze drying, also known as lyophilization, is gaining in popularity as an alternative technique [14]. Acar et al. (2011) compared lyophilized saffron with sun-dried saffron. The safranal content of the lyophilized sample was five times higher than that of the sun-dried saffron [15]. Lyophilization protects thermolabile compounds and minimizes exposure to oxygen, which can produce undesirable oxidation reactions; however, lyophilization can result in loss of volatiles and is also a relatively expensive drying process [16].

While several studies have examined the chemical composition and beneficial effects of saffron petals, the impact of drying on the preservation of bioactive compounds has not been thoroughly investigated. Therefore, this research aimed to study the kinetic changes of valuable compounds of saffron petals, such as carotenoids, anthocyanins, and flavonols, during three different drying processes. For this purpose, saffron petals were subjected to oven-drying, vacuum evaporation, and lyophilization and were analyzed by LC–QTOF MS/MS for metabolite annotation. Temperature and drying time were evaluated as variables with a critical influence on the content of bioactive compounds. Fresh petals were also extracted for reference.

## 2. Materials and Methods

### 2.1. Samples, Reagents, and Solvents

Five hundred grams of fresh petals were provided by the PDO La Mancha Saffron (Camuñas, Toledo, Spain). Petals were hand-picked at the end of October 2022 and immediately frozen at −80 °C. Then, petals were homogenized with a mortar using liquid nitrogen to minimize the degradation of components. 1-g aliquots were stored at −80 °C until drying procedures were applied. After drying, it was powdered with mortar to achieve a millimeter diameter. LC-grade solvents were used for sample preparation and mobile chromatographic phases. Methanol, acetonitrile, and formic acid were sourced from Fisher Scientific (Madrid, Spain), while acetone and chloroform were acquired from Scharlab (Barcelona, Spain). Deionized water (18 MΩ·cm) was sourced from a Milli-Q water purification system manufactured by Millipore (Bedford, MA, USA). 

Coumaric acid, crocin (cis/trans mixture), safranal, quercetin, and hesperidin standards were purchased from Sigma-Aldrich (St. Louis, MO, USA). Kaempferol was purchased from Extrasynthese (Genay, France); and cyanidin-3-glucoside, delphinidin-3-glucoside, kaempferol-neohesperidoside, quercetin-3-glucoside, kaempferol-7-glucoside, and isorhamnetin-3-glucoside were purchased from Cymit Química (Barcelona, Spain).

### 2.2. Apparatus and Instruments

A lyophilizer Lyoquest HT40 Benjer electronics from Telstar (Barcelona, Spain), equipped with thermal plates, was used both for lyophilization and vacuum evaporation. Oven Mod. 10 PR/300 series 8B, which allows forced ventilation, was used for air-drying (Hobersal, Barcelona, Spain). A vortex shaker from IKA (Wilmington, NC, USA), and an Eppendorf ThermoMixer^®^ C (Eppendorf, Madrid, Spain) were used for extraction. 

An Agilent 1200 Series liquid chromatography (LC) system (Agilent Technologies, Palo Alto, CA, USA) equipped with a Zorbax Eclipse Plus C18 HD reversed-phase analytical column (3.0 × 150 mm, 1.8 µm) and a guard column (3.0 × 5 mm, 1.8 µm) was used for chromatographic separation. Detection was performed with a 6540 quadrupole time-of-flight mass spectrometer (QTOF MS/MS) from Agilent Technologies (Santa Clara, CA, USA), while data acquisition and both qualitative and quantitative analyses were conducted using Agilent MassHunter Workstation software (version V-B.09). 

### 2.3. Saffron Petals Drying

Sample processing included the evaluation of three different drying processes: lyophilization, oven-drying, and vacuum evaporation. Lyophilization was performed at −50 °C and samples were weighed and analyzed after 8, 24, 32, and 48 h of drying. On the other hand, samples were oven-dried at two different temperatures, 40 °C, and 60 °C; samples were analyzed at 4, 8, 24, 32, and 48 h. Finally, for vacuum evaporation, the process was carried out in 5 cycles at 2 different temperatures, 25 °C and 50 °C. Each cycle has a duration of 58 min consisting of 3 min of condenser freezing at −50 °C, 15 min of vacuum (below 10 mbar), 20 min of heating at 25 °C or 50 °C, and 20 min of vacuum (final pressure at 0.05 mbar). Each experiment was performed in triplicate. Supplementary Table S1 lists the experiments programmed according to the defined variables. Dried samples were stored at −80 °C until they were subjected to extraction [16].

The extraction solvent comprised a mixture of methanol, acetone, chloroform, and water (70:10:10:10, *v*/*v*), acidified with 1% formic acid and with hesperidin at a concentration of 5 mg L^−1^ as the internal standard (IS). This extractant has been proven to effectively extract metabolites across broader polarities [17]. Each extraction was performed by adding 2 mL of extractant to 1 g of dried petals. The mixture was then shaken for 30 min at 30 °C and 1000 rpm. This extraction process was repeated twice, and the resulting 6 mL extract was filtered through a 0.2 μm nylon filter prior to the injection into the LC–QTOF MS/MS. Three replicates of fresh petals were also extracted for reference.

### 2.4. LC–QTOF MS/MS Analysis of Saffron Extracts 

Extracts were analyzed by LC–MS/MS in high resolution using data-independent acquisition (DIA) mode. The analysis was performed on an Agilent Technologies LC–QTOF MS/MS 1200 series. Chromatographic separation was performed using water (mobile phase A) and acetonitrile (mobile phase B), each with 0.1% formic acid as the ionizing agent. The elution gradient was as follows: from 0 to 1 min, phase B was set at 4%; from 1 to 6 min, phase B increased linearly from 4% to 40%; from 6 to 10 min, phase B further increased from 40% to 100%. Phase B was held at 100% from 10 to 20 min to ensure complete elution of all sample components. The column was then re-equilibrated to starting conditions over 13 min before the next run. The chromatographic flow rate was set at 0.25 mL/min, with a 2 µL injection volume. Electrospray ionization (ESI) settings in both positive and negative modes included a nebulizer gas pressure of 40 psi; drying gas (N_2_) flow rate and temperature of 12 L/min and 325 °C, respectively; capillary voltage of ±3.5 kV; and voltages of 130, 65, and 750 V for Q1, skimmer, and octopole, respectively.

For DIA, data were acquired at a rate of 5 spectra per second across 3 channels, each using variable collision energies (CE) set at 0, 20, and 40 eV, with a cycle time of 1 s per channel, resulting in a total cycle time of 3 s. All channels covered an acquisition range of 40–1100 *m*/*z*. Accurate *m*/*z* measurement on the 0 eV channel (non-fragmented) was maintained in all analyses by continuous internal calibration, using signals at *m*/*z* 112.9856 (trifluoroacetic acid anion) and 1033.9881 (HP-921, hexakis(1H,1H,3H-tetrafluoropropoxy)phosphazine).

### 2.5. Data Processing and Statistical Analysis 

Metabolite annotation was performed using MetaboMSDIA, a tool previously developed by the authors [18] (https://github.com/MonicaCalSan/MetaboMSDIA, accessed on 9 September 2024) for data analyses using DIA mode. MetaboMSDIA is an informatic tool that combines the MS1 data stored in different acquisition channels at different collision energies to obtain multiplexed MS2 spectra in a single run. Briefly, the tool extracts the information of all channels to obtain chromatographic peaks at the different collision energies. The information is filtered to remove isotopically associated ions (isotopic distribution), adducts, and in-source fragments formed in the ESI unit. Then, find association patterns of coeluting chromatographic peaks among all ions scanned in the 0 eV channel (considered precursor ions) and 20 eV and 40 eV (both considered product ions) and create multiplexed MS2 spectra for all precursor ions which have been used for metabolites annotation, comparing with those stored in a home-made MS/MS database containing analytical standards, also tentatively identified metabolites based on their characteristic fragmentation were included. 

Quantitative analysis was conducted by creating calibration models for the available standards within a concentration range of 1–20 µg/mL. Quantification was performed for those compounds without available standards by applying calibration models of structurally similar compounds. 

MetaboAnalyst 6.0 (https://metaboanalyst.ca/, accessed on 16 September 2024) and Statgraphics Centurion XVIII software (The Plains, VA, USA) were used for statistical analysis. Quantitative data were normalized by logarithmic transformation and scaled. Tuckey post-hoc analysis of variance (ANOVA) at the 95% confidence level tested variability between groups of samples. Principal component analysis (PCA) was tested to identify differences in the drying process.

### 2.6. DPPH Radical Scavenging Activity

The DPPH radical scavenging activity was evaluated by mixing 100 µL of sample (either crude extract or fraction) with 900 µL of 20 mg/L DPPH solution in MeOH. The mixture was stirred for 30 min at room temperature, and the absorbance was measured at 517 nm. A reference solution was prepared following the same procedure using MeOH instead of the sample. The antiradical scavenging potential was expressed as the percentage of decoloration of DPPH solution by the following equation:

$$\%Radical\;scavenging=\left[1-\frac{A_{s}}{A_{R}}\right]*100$$

## 3. Results

### 3.1. Annotation of Bioactive Compounds in Saffron Petals

After processing the experimental data obtained by LC–QTOF MS/MS, a total of 22 bioactive compounds were annotated in petal extracts. These were coumaric acid, safranal, *cis* and *trans* crocin, and flavonoids, including 4 anthocyanins and 15 flavonols. The last group included kaempferol, quercetin, and their derivatives. Table 1 summarizes the main parameters (neutral mass, formula, retention time, detected adduct, precursor ion, and product ions) for identifying these compounds. The identification of 12 metabolites was confirmed by injection of analytical standards. Additional annotations were done by searching experimental MS information and MS/MS fragmentation patterns in online databases and scientific literature.

Crocins, picrocrocin, and safranal are the major secondary metabolites in saffron plants. Crocins are a family of glycosyl esters of crocetin (Supplementary Figure S1) that differ in their sugar moieties and the *cis/trans* isomeric configuration. They are the main pigment of saffron stigmas but have also been identified in petals [19]. We identified in petals both the *cis* and *trans* isomeric forms in petals. Both isomers showed two main fragments in negative mode (*m*/*z* 651.278, 327.168) due to the successive losses of the two gentiobiose substituents in their structure (324.106 Da), which, according to the literature, are among the most abundant glycosides in saffron petals [20]. Safranal was detected only in positive mode. 

On the other hand, 4-coumaric acid (or *p*-coumaric acid) was identified in negative ionization mode. Its importance lies in the fact that it is the precursor of many natural products, such as flavonoids, which possess antioxidant properties [21]. Furthermore, flavonoids (mainly anthocyanins, flavonols, and flavones) are one of the most important classes of natural enzyme inhibitors [22]. The petals and sepals of *C. sativus* are a rich source of glycosylated flavonols [23]. The flavonols identified in this study were kaempferol, quercetin, and their glucosides as mono- or di-glucosides. Kaempferol compounds showed the protonated aglycone fragment at *m*/*z* 287.054 and the deprotonated aglycone fragment at *m*/*z* 284.034 and 285.037. The presence of a fragment ion at *m*/*z* 284.034 corresponding to a kaempferol moiety, instead of the expected ion at *m*/*z* about 285.037, has been described as a characteristic product ion for kaempferol-3-glucoside ([M-163-H]^−^), but not for kaempferol-7-glucoside [24]. Similar to kaempferol, quercetin derivatives were identified by forming base peak ions at *m*/*z* 301.035 [M−H]^−^ and *m*/*z* 303.0508 [M+H]^+^.

Anthocyanins are molecules responsible for the color of petal extracts, and they can be used as a natural colorant in the food industry [25]. Delphinidin-3-glucoside, delphinidin-3,5-diglucoside, cyanidin-3-glucoside, and cyanidin-3,5-diglucoside were detected in the petal extracts. They appeared in the positive mode as [M]^+^, and [M+H]^+^ for delphinidin-3,5-diglucoside. These anthocyanins were confirmed by the appearance of the characteristic production of the aglycones cyanidin (at *m*/*z* 287.059) and delphinidin (*m*/*z* 303.049) after the loss of glucose. One limitation in annotating anthocyanins by MS/MS without confirmation by analytical standards is that they are naturally positively charged deprotonated ions that may have the same *m*/*z* value as some flavonoids in positive ionization mode. For example, quercetin and delphinidin have the same precursor ion in positive mode, *m*/*z* 303.049 [M+H]^+^ and [M]^+^, respectively, but the ion structure differs. One strategy to ensure the assignment is to complement this result with that obtained in negative ionization mode. Anthocyanins are not detected in negative mode; therefore, flavonoids and anthocyanins can be distinguished.

### 3.2. Changes in the Moisture Content During Drying Kinetics 

**Figure 1:**
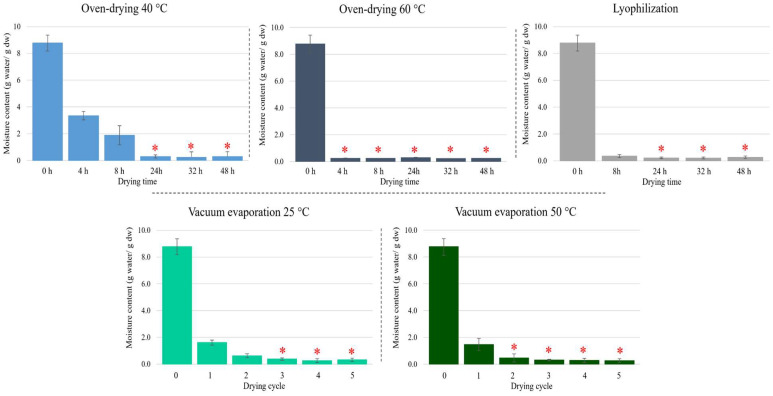
Moisture content (g water/g dry weight) versus time or processing cycles for each drying process used in this research. Asterisks point out indicate no significant differences (Tukey HSD, *p*-value < 0.05).

The moisture content of the petals determined by the reference AOAC method was 89.8%, which means that 1 g of fresh petals yielded 0.102 g dry weight (dw). This is equivalent to 8.8 g water/g dw. In this research, alternative drying was performed using three different processes: oven-drying, vacuum evaporation, and lyophilization. The moisture content versus time or processing cycles is shown in Figure 1, which also shows the significance (Tukey HSD, *p*-value < 0.05). In oven-drying, temperature plays a key role, since at 40 °C the process required 24 h to achieve the lowest moisture content (≈0.3 g water/g dw), with no significant differences (*p*-value < 0.05) compared to longer times. At 4 h and 8 h, the moisture contents were particularly relevant with values of 3.3 and 1.9 g water/g dw, respectively, corresponding to 56 and 70% moisture loss, respectively. On the other hand, oven-drying at 60 °C showed no significant changes after 4 h with a moisture content of 0.25 g water/g dw (Figure 1). Concerning lyophilization, the first control point for analysis was at 8 h, the moisture content being 0.36 g water/g dw. However, the lowest moisture content (0.22 g water/g dw) was found at 24 h, and no significant changes were observed in subsequent measurements (Figure 1).

Vacuum evaporation was the fastest drying method under study. At 25 °C, the lowest moisture content (0.32 g water/g dw) was reached after 3 drying cycles (approximately 3 h), while at 50 °C, only 2 cycles were required to reach 0.18 g water/g dw; in both cases, no significant changes were observed with additional drying cycles (Figure 1). The protocols can be compared regarding residual moisture content, ranging from 0.18 to 0.30 g water/g dw (Supplementary Table S2). Thus, temperature improved petal drying in both oven and vacuum evaporation. Furthermore, vacuum evaporation provided the most efficient results, as the drying process was completed in about 2 h when the temperature increased to 50 °C.

### 3.3. Evolution of Bioactive Compounds During Drying Kinetics 

**Figure 2:**
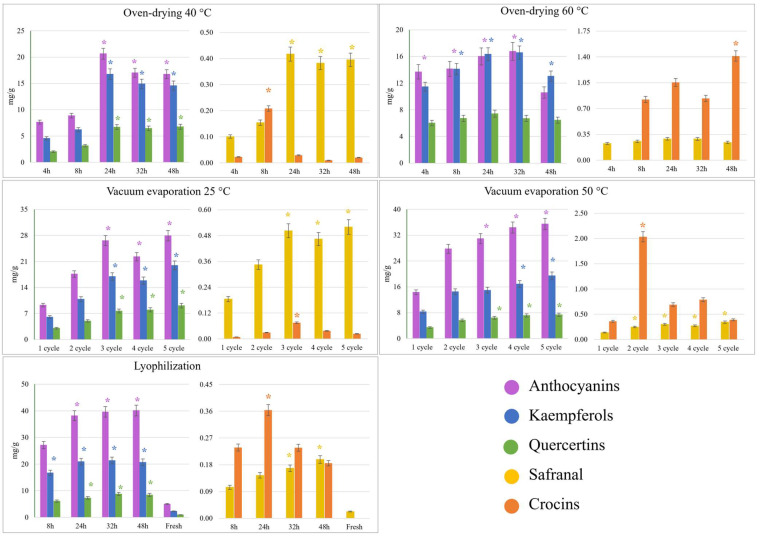
Concentration expressed as mg/g (dry weight) of the main families of bioactive compounds versus drying time or cycles for each process. Asterisks indicate no significant differences (Tukey HSD, *p*-value < 0.05).

Quantitative analysis was performed by preparing calibration models in the 0.1–20 g/mL concentration range. For those compounds without standard, quantitation was performed by applying calibration models obtained with structurally similar compounds (Supplementary Table S3). We monitored the concentration of the main families of bioactive compounds, namely, anthocyanins, flavonoids, crocins, and safranal, in extracts obtained after applying the drying protocols (Figure 2). The drying temperature significantly influenced the composition of the extracts. Thus, oven-drying at 40 °C provided the highest concentrations of bioactive compounds after 24 h (*p*-value < 0.05), except for crocins, which showed maximum concentration after 8 h. Then, crocins were significantly reduced at longer times. Anthocyanins were the most concentrated family, with a maximum of 20.7 mg/g, followed by kaempferol derivatives (16.8 mg/g) and quercetin derivatives (6.7 mg/g). The maximum concentration of minor components was 0.21 mg/g for crocins and 0.42 mg/g for safranal. On the other hand, the concentration of anthocyanins and kaempferol derivatives at 60 °C was similar during the first 32 h (≈15 mg/g for anthocyanins and kaempferol derivatives). However, anthocyanins and kaempferol derivatives significantly decreased at 48 h to 10.6 and 13.0 mg/g, respectively. The maximum concentration was reached after 8 h of drying and no significant changes were observed thereafter. Concerning crocins, an increase in concentration was observed during drying from 0.8 mg/g at 8 h to 1.4 mg/g at 48 h. In this experiment, no significant differences were observed in the concentration of quercetin and safranal. The differences in anthocyanin content between oven-drying at 40 °C (20.7 mg/g) and 60 °C (16 mg/g) are noteworthy. However, the concentration of flavonoids was similar. The effect of time and temperature on the stability of anthocyanins has been previously reported, and it has been shown that there is a degradation of these compounds when the temperature is higher and longer [26]. 

The opposite effect was observed when drying by vacuum evaporation at 25 °C and 50 °C. In both processes, we observed a gradual increase in the concentration of major families reaching their maximum concentration after three cycles, except for kaempferols at 50 °C, which reached the maximum concentration after four cycles; however, it is important to note that the concentration of kaempferols and quercetins was similar after three cycles; however, anthocyanins were higher at 50 °C than at 25 °C, 34.3 mg/g versus 26.6 mg/g, respectively. This result suggests that the above-mentioned degradation by time and temperature could not be the only factor affecting the stability of anthocyanins, but also the oxygen present in the environment is key for this degradation since, in these experiments, the vacuum pressure was 0.05 mbar. On the other hand, safranal and crocins reached their maximum concentration at 25 °C after two cycles (0.5 mg/g and 0.08 mg/g, respectively), whereas at 50 °C only two cycles were necessary (0.25 mg/g and 2 mg/g, respectively). Considering the oven-drying results, it seems that the temperature strongly influences the final concentration of crocins since, in both drying methods, the content of crocins was increased at higher temperatures, regardless of the oxygen concentration during the process. 

Finally, in the case of lyophilization, the maximum concentration of bioactive compounds (anthocyanins, 39 mg/g; kaempferols, 20 mg/g; quercetins, 8.5 mg/g; and crocins, 0.36 mg/g) was obtained after processing for 24 h, except for safranal (0.2 mg/g) which required 32 h (Figure 2). These results showed that lyophilization allowed obtaining extracts with a higher content of anthocyanins (the most abundant bioactive in saffron petals) and supported the theory of the combined effect of low temperature and oxygen content since this process was carried out at −50 °C and 0.05 mbar. Also, the content of flavonoids was higher in these extracts, which may be due to the slow kinetics of degradation reactions under freezing conditions (−50 °C). In addition, the influence of high temperatures to increase the crocin content was confirmed since vacuum evaporation at 50 °C and oven-drying at 60 °C gave a higher content of this metabolite (2 mg/g and 1.4 mg/g, respectively) than lyophilization at −50 °C (0.36 mg/g).

In addition to the content of bioactive compounds, we measured the antioxidant activity of the extracts obtained under optimal conditions. In concordance with the total content of bioactive compounds, the extracts obtained after lyophilization, oven-drying at 60 °C and vacuum evaporation at 50 °C provided the maximum antioxidant activity (46.9, 44.5 and 44.2%, respectively) with no significant differences (*p*-value < 0.05) among them. On the other hand, the extracts obtained after vacuum evaporation at 25 °C and oven-drying at 40 °C showed significantly less activity (38.1% and 37.9%, respectively) than those obtained at higher temperatures. The high activity showed for the lyophilization extracts can be explained by the higher content of flavonoids while concerning drying methods at higher temperatures (oven-drying at 60 °C and vacuum evaporation at 50 °C), the contents of crocins and flavonoids could explain the high antioxidant activity.

### 3.4. Influence of Drying Methods on the Main Bioactive Families Isolated from Saffron Petals

Dried samples contained crocins, safranal, anthocyanins, and flavonols with minimum and maximum values ranging from <LOQ–2.04, 0.10–0.52, 7.7–40.1, and 6.6–30.1 mg/g, respectively. The concentrations measured in fresh petals were significantly lower (<LOQ, 0.02, 5.0, and 3.3 mg/g, respectively). This can be explained by the presence of water in petals, which interferes with extraction, in addition to the fact that it can promote hydrolysis reactions that would cause the degradation of analytes.

**Figure 3:**
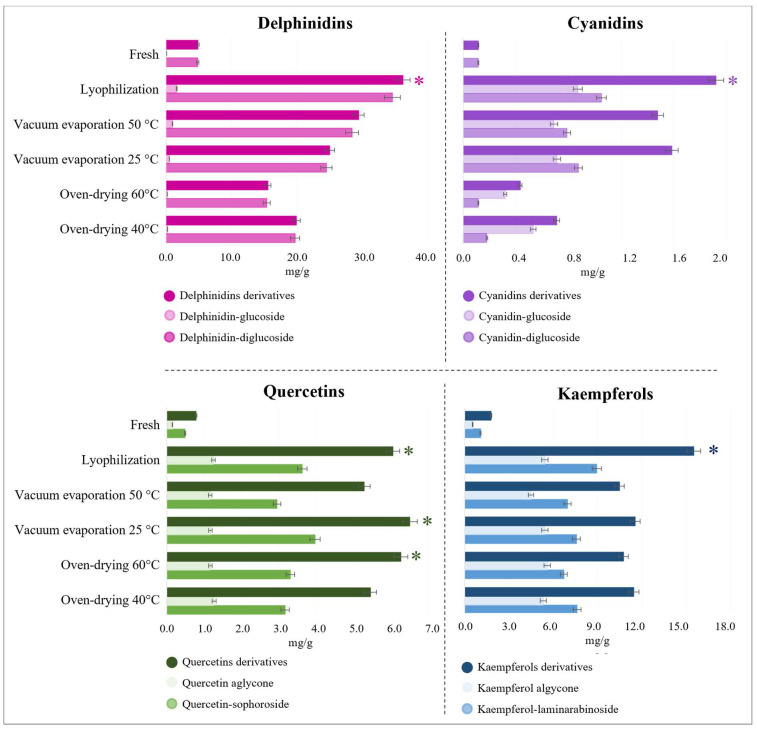
Mean concentrations expressed as mg/g (dry weight) in fresh and dried petals under suited drying conditions by oven-drying, vacuum evaporation, and lyophilization. Bars with an asterisk indicate that the concentration of this metabolite was significantly higher.

The anthocyanin family was the most abundant in saffron petals, with a high content of delphinidin derivatives (approximately 95%). For example, in lyophilized samples, the concentration of delphinidin-3,5-diglucoside represented 90.7% of the total anthocyanins present in the petals, while 4.3% corresponded to delphinidin-3-glucoside. In addition, two cyanidin derivatives were identified, representing only 5% of the total anthocyanins in the extracts. These proportions were almost constant for all drying methods, suggesting that glycosidic bond cleavage reactions did not occur under the conditions studied since no aglycone anthocyanins were identified. Figure 3 shows each drying method’s maximum total and major anthocyanin content. 

Regarding kaempferols, the aglycone form represented 23% and 26% of the total kaempferol derivatives in lyophilized and fresh samples, respectively, whereas, in samples dried with temperatures above 25 °C, this proportion ranged from 30% to 34%, being superior for oven-drying. Nevertheless, the relative content of kaempferol-laminarabinoside in all samples was similar in an interval of 42% to 46%. Kaempferol and kaempferol derivatives represented approximately 31% of the total compounds. Zeka et al. (2015) previously reported the high concentration of kaempferol compounds in saffron petals, which show antioxidant activity and can be used as a food supplement [20]. On the other hand, the proportion of quercetin aglycones did not show the same behavior as kaempferol; in this case, the proportion of aglycones did not show significant differences between drying methods, ranging from 16% to 18%. Quercetin-3-sophoroside represented almost 50% of the quercetin conjugates in all samples (Figure 3). The high flavonoid content in dried saffron petals shows that this residue could be used as an asset to provide anthocyanin and flavonol contents for food and non-food industrial applications [27].

### 3.5. Influence of Drying on Bioactive Compounds Profile

**Figure 4:**
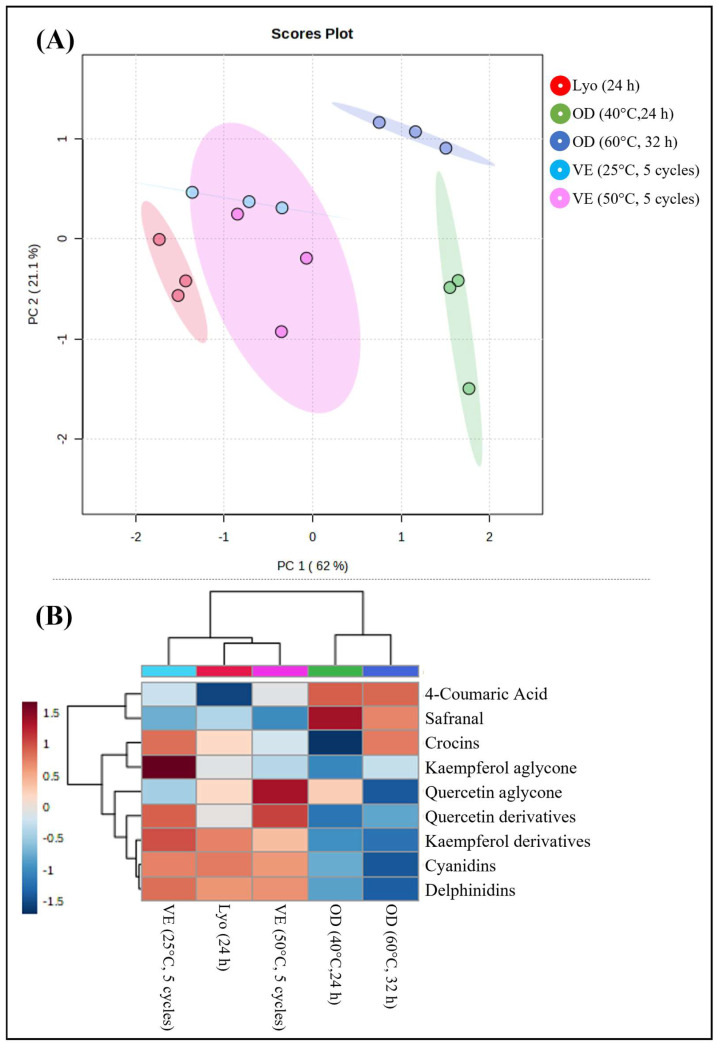
Principal components analysis (**A**) and heatmap (**B**) show the distribution of drying processes according to the concentration of bioactive compounds. Lyo, lyophilization; OD, oven-drying and VE, vacuum evaporation.

After evaluating the drying kinetics, the optimal conditions were selected for each process. These were as follows: oven-drying at 40 °C for 24 h; oven-drying at 60 °C for 32 h; lyophilization for 24 h; and three cycles of vacuum evaporation for both temperatures, 25 and 50 °C. Principal component analysis (PCA) showed discrimination as a function of the drying process (Figure 4A). 

Two groups can be distinguished along component 1 (PC1), corresponding to those carried out under vacuum conditions (lyophilization and vacuum evaporation at 25 and 50 °C) and those subjected to oven-drying. The temperature effect on the composition of the extracts seems to be more critical for oven-drying than vacuum evaporation, which supports the influence of changes caused by the presence of oxygen. The combination of PC1–PC2 components explained 83.1% of the total variability, which allowed us to conclude that the petal profile strongly depends on the drying process. A Heatmap analysis (Figure 4B) allows the visualization of changes in compounds related to the drying process. The concentrations of 4-coumaric acid, safranal, and crocins were maximum for oven-drying, especially at 60 °C, while the contents of anthocyanins and flavonoids were maximum for vacuum drying protocols. This effect was explained by the preservation of anthocyanins under vacuum conditions and the antioxidant properties of flavonoids, which could be affected by oxidation [28].

## 4. Conclusions

Saffron petals are a valuable source of bioactive compounds such as anthocyanins or flavonoids, especially kaempferol and quercetin derivatives. This study has shown that dehydration of the sample before extraction is key to increasing the extraction yield. However, the choice of the drying method is also crucial for the function of the compounds to be obtained. Thus, the combined effect of the low temperature and oxygen concentration (0.05 mbar for all procedures under vacuum) was found to increase the anthocyanin content or the suitability of the high-temperature drying to maximize the crocins in the extract. These results show the influence of different drying methods on the extraction of metabolites from saffron petals from a chemical point of view. Thus, lyophilization was the most suitable method to obtain higher contents of all metabolites, except for crocins, which were higher over 50 °C. However, a cost-benefit study is required before selecting the most suitable method, since lyophilization required maintaining both the sample and the condenser below −50 °C for 24 h to obtain the mentioned results.

## Figures and Tables

**Table 1:** List of compounds with the main parameters (neutral mass, formula, retention time, adduct, precursor ion, and fragment ions) used for annotation.

Compound	Mass	Molecular Formula	RT (min)	Ion/ Adduct	Precursor Ion (*m*/*z*)	Main Product Ions (*m*/*z*)		
Safranal *	150.105	C_10_H_14_O	9.62	[M+H]^+^	151.110	91.053	81.070	67.054
*trans*-Crocin *	976.375	C_44_H_64_O_24_	9.95	[M−H]^−^	975.376	651.261	327.157	
*cis*-Crocin *	976.375	C_44_H_64_O_24_	12.56	[M−H]^−^	975.376	651.261	327.157	
4-Coumaric acid *	164.048	C_9_H_8_O_3_	10.79	[M−H]^−^	163.040	123.277	81.703	50.007
Anthocyanins								
Delphinidin-3,5-diglucoside	626.149	C_27_H_30_O_17_	7.55	[M+H]^+^	627.157	465.100	303.049	
Cyanidin-3,5-diglucoside	611.161	C_27_H_31_O_16_	7.77	[M]^+^	611.161	449.112	287.059	166.089
Cyanidin-3-glucoside *	449.108	C_21_H_21_O_11_	9.70	[M]^+^	449.108	287.056	258.050	213.056
Delphinidin-3-glucoside *	465.102	C_21_H_21_O_12_	10.29	[M]^+^	465.102	303.051	257.045	229.050
Flavonols								
Kaempferol & derivatives								
Kaempferol-3-sophoroside	610.154	C_27_H_30_O_16_	8.16	[M+H]^+^	611.162	449.105	287.054	145.050
Kaempferol-6″-malonyl-glucoside-glucoside	696.154	C_30_H_32_O_19_	8.34	[M+H]^+^	697.157	449.106	287.054	145.049
Kaempferol-3-glucoside *	448.093	C_21_H_20_O_11_	8.59	[M−H]^−^	447.093	284.034	255.303	227.036
Kaempferol-laminarabinoside	610.153	C_27_H_30_O_16_	9.21	[M−H]^−^	609.149	284.032	225.030	151.003
Kaempferol-coumaroylrutinoside	740.195	C_36_H_36_O_17_	10.00	[M+HCOO]^−^	785.195	623.149	315.047	285.039
Kaempferol-neohesperidoside *	594.155	C_27_H_30_O_15_	10.41	[M−H]^−^	593.142	285.037	284.032	255.027
Kaempferol-3-(6″-acetylglucoside)-7-glucoside	652.164	C_29_H_32_O_17_	10.50	[M−H]^+^	653.171	491.121	287.057	
Kaempferol-3-(6″-acetylglucoside)	490.111	C_23_H_22_O_12_	11.14	[M−H]^−^	489.107	284.031	255.034	227.033
Kaempferol *	286.049	C_15_H_10_O_6_	14.19	[M+H]^+^	287.056	213.053	153.016	121.027
Quercetin & derivatives								
Quercetin-isopropylglucoside-glucoside	684.190	C_30_H_36_O_18_	8.11	[M−H]^−^	683.166	463.089	301.037	125.022
Quercetin-3-sophoroside	626.148	C_27_H_30_O_17_	8.63	[M−H]^−^	625.143	463.086	301.033	
Quercetin 3-glucoside *	464.095	C_21_H_20_O_12_	9.83	[M−H]^−^	463.0907	300.027	271.025	255.030
Isorhamnetin-3-glucoside *	478.112	C_22_H_22_O_12_	10.15	[M−H]^−^	477.105	314.042	271.023	243.020
Rutin *	610.142	C_27_H_30_O_16_	11.36	[M−H]^−^	609.138	463.088	301.035	285.040
Quercetin *	302.043	C_15_H_10_O_7_	13.47	[M+H]^+^	303.051	229.054	153.021	137.026

* Identification confirmed by analytical standards; RT, retention time.

## Data Availability

The original contributions presented in the study are included in the article and Supplementary Material, further inquiries can be directed to the corresponding author.

## Supplementary Materials

The following supporting information can be downloaded at: https://www.mdpi.com/article/10.3390/foods13233724/s1, Figure S1. Chemical structures of crocin, safranal and flavonoids (flavonols and anthocyanins) identified in *Crocus sativus* petals; Table S1. The experimental design used for evaluation of different drying techniques; Table S2. Residual moisture content in the saffron petals dried with different methods; Table S3. Concentrations (mean ± SD, expressed as mg/g) of main bioactive compounds in fresh and dried petals following different drying processes (OD, oven-drying; VE, vacuum evaporation and Lyo, lyophilization). Maximum, minimum, and mean value concentrations are also listed. LOQ, out of quantitation limit.
