# Supplementary material for: Drying of Saffron Petals as a Critical Step for the Stabilization of This Floral Residue Prior to Extraction of Bioactive Compounds
Source: Foods. 2024 Nov 21;13(23):3724. doi: 10.3390/foods13233724 (PMC11640713; doi:10.3390/foods13233724)
Supplement: Supplementary file 1 [file foods-13-03724-s001.zip › foods-3297621-supplementary.pdf]

Supplementary information

Drying of Saffron Petals as a Critical Step for the Stabilization of This Floral Residue Prior to Extraction of Bioactive Compounds

Inmaculada Criado-Navarro [1,2,3,4], Francisco Barba-Palomeque [1,2,3,4], Pedro Pérez-Juan [5], Carlos A. Ledesma-Escobar [1,2,3,4,*] and Feliciano Priego-Capote [1,2,3,4]

[1] Department of Analytical Chemistry, University of Córdoba, 14071 Córdoba, Spain; q12crnai@uco.es (I.C.-N.); qa2bapaf@uco.es (F.B.-P.); q72prcaf@uco.es (F.P.-C.)
[2] Chemical Institute for Energy and Environment (iQUEMA), University of Córdoba, 14071 Córdoba, Spain
[3] Maimónides Institute of Biomedical Research (IMIBIC), Reina Sofía University Hospital, University of Córdoba, 14004 Córdoba, Spain
[4] CIBER of Frailty and Healthy Ageing (CIBERFES), Carlos III Health Institute, 28029 Madrid, Spain
[5] Regulatory Council Foundation of the Protected Designation of Origin Azafrán de La Mancha, 45720 Toledo, Spain; gerencia@doazafrandelamancha.com
* Correspondence: z32leesc@uco.es

Supplementary Table S1. The experimental design used for evaluation of different drying techniques.

	Temperature (ºC)	Time (h)	Cycle	Sample	Extract
**Lyophilization**	−50	8		frozen	Lyo (8 h)
	−50	24		frozen	Lyo (24 h)
	−50	32		frozen	Lyo (32 h)
	−50	48		frozen	Lyo (48 h)
**Oven-drying**	40	4		thawed	OD (40°C, 4 h)
	40	8		thawed	OD (40°C, 8 h)
	40	24		thawed	OD (40°C, 24 h)
	40	32		thawed	OD (40°C, 32 h)
	40	48		thawed	OD (40°C, 48 h)
	60	4		thawed	OD (60°C, 4 h)
	60	8		thawed	OD (60°C, 8 h)
	60	24		thawed	OD (60°C, 24 h)
	60	32		thawed	OD (60°C, 32 h)
	60	48		thawed	OD (60°C, 48 h)
**Vacuum evaporation**	25		1	thawed	VE (25°C, 1 cycle)
	25		2	thawed	VE (25°C, 2 cycle)
	25		3	thawed	VE (25°C, 3 cycle)
	25		4	thawed	VE (25°C, 4 cycle)
	25		5	thawed	VE (25°C, 5 cycle)
	50		1	thawed	VE (50°C, 1 cycle)
	50		2	thawed	VE (50°C, 2 cycle)
	50		3	thawed	VE (50°C, 3 cycle)
	50		4	thawed	VE (50°C, 4 cycle)
	50		5	thawed	VE (50°C, 5 cycle)

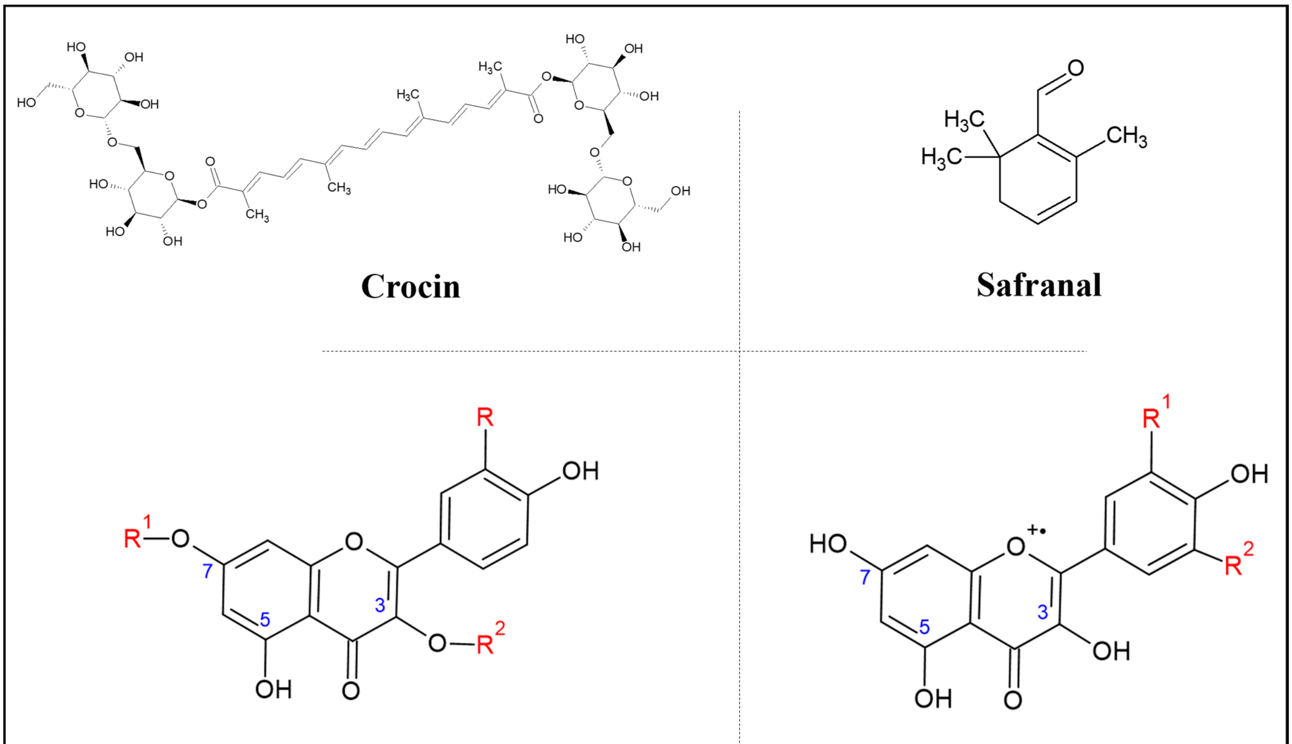

Flavonols	R	R^1	R^2
**Kaempferol**	H	H or Glucosyl	H or Glucosyl
**Quercetin**	OH	H or Glucosyl	H or Glucosyl
**Isorhamnetin**	OCH_3	H or Glucosyl	H or Glucosyl

Anthocyanins	R^1	R^2
**Delphinidin**	OH	OH
**Cyanidin**	OH	H
**Malvidin**	OCH_3	OCH_3

Supplementary Figure S1. Chemical structures of crocin, safranal and flavonoids (flavonols and anthocyanins) identified in *Crocus sativus* petals.

Supplementary Table S2. Residual moisture content in the saffron petals dried with different methods.

	Moisture content (g water/g dw)
Oven-drying 40 °C	0.30
Oven-drying 60 °C	0.24
Lyophilization	0.23
Vacuum evaporation 25 °C	0.27
Vacuum evaporation 50 °C	0.18
Reference AOAC method	0.22

Supplementary Table S3. Concentrations (mean±SD, expressed as mg/g) of main bioactive compounds in fresh and dried petals following different drying processes (OD, oven-drying; VE, vacuum evaporation and Lyo, lyophilization). Maximum, minimum, and mean value concentrations are also listed. LOQ, out of quantitation limit.

Sample	4-Coumaric Acid	Crocins	Safranal	Anthocyanins	Flavonols
OD (40°C, 4 h)	0.13 ± 0.05	0.02 ± 0.04	0.1 ± 0.01	7.7 ± 0.7	6.6 ± 0.14
OD (40°C, 8 h)	0.18 ± 0.06	0.21 ± 0.36	0.15 ± 0.02	8.9 ± 1.2	9.4 ± 1.8
OD (40°C, 24 h)	0.26 ± 0.1	0.03 ± 0.03	0.42 ± 0.03	20.7 ± 4.0	23.5 ± 4.8
OD (40°C, 32 h)	0.24 ± 0.02	LOQ	0.38 ± 0.07	17.0 ± 2.0	21.4 ± 2.9
OD (40°C, 48 h)	0.26 ± 0.08	0.02 ± 0.04	0.4 ± 0.06	16.8 ± 4.0	21.4 ± 4.7
OD (60°C, 4 h)	0.45 ± 0.12	LOQ	0.23 ± 0.04	13.7 ± 2.4	17.5 ± 1.3
OD (60°C, 8 h)	0.2 ± 0.1	0.82 ± 0.6	0.25 ± 0.05	14.1 ± 1.2	20.8 ± 2.0
OD (60°C, 24 h)	0.26 ± 0.09	1.06 ± 1.24	0.29 ± 0.07	16.0 ± 4.0	23.8 ± 3.9
OD (60°C, 32 h)	0.22 ± 0.05	0.84 ± 0.72	0.29 ± 0.08	16.8 ± 5.0	23.3 ± 5.4
OD (60°C, 48 h)	0.28 ± 0.12	1.41 ± 0.63	0.24 ± 0.02	10.6 ± 1.5	19.5 ± 2.6
VE (25°C, 1 cycle)	0.05 ± 0.03	0.01 ± 0.02	0.19 ± 0.01	9.3 ± 0.9	9.1 ± 1.5
VE (25°C, 2 cycle)	0.04 ± 0.03	0.03 ± 0.03	0.34 ± 0.06	17.6 ± 4.1	15.9 ± 2.2
VE (25°C, 3 cycle)	0.05 ± 0	0.08 ± 0.02	0.5 ± 0.16	26.6 ± 10.8	24.6 ± 6.7
VE (25°C, 4 cycle)	0.07 ± 0.03	0.04 ± 0.06	0.46 ± 0.07	22.3 ± 4.2	23.9 ± 3.5
VE (25°C, 5 cycle)	0.08 ± 0.02	0.02 ± 0.04	0.52 ± 0.1	27.9 ± 8.6	29.1 ± 7.1
VE (50°C, 1 cycle)	0.02 ± 0.01	0.36 ± 0.5	0.14 ± 0.02	14.3 ± 0.7	11.7 ± 1.6
VE (50°C, 2 cycle)	0.03 ± 0.01	2.04 ± 0.55	0.25 ± 0.03	27.8 ± 6.5	20.2 ± 3.0
VE (50°C, 3 cycle)	0.02 ± 0.01	0.69 ± 0.17	0.3 ± 0.02	30.9 ± 1.8	21.4 ± 1.6
VE (50°C, 4 cycle)	0.02 ± 0.01	0.79 ± 0.3	0.27 ± 0.02	34.3 ± 2.1	24.0 ± 1.8
VE (50°C, 5 cycle)	0.03 ± 0	0.39 ± 0.55	0.34 ± 0	35.4 ± 2.6	26.8 ± 1.7
Lyo (8 h)	LOQ	0.24 ± 0.02	0.1 ± 0.01	27.1 ± 3.8	22.8 ± 3.5
Lyo (24 h)	LOQ	0.36 ± 0.24	0.14 ± 0.02	38.2 ± 3.9	28.2 ± 1.7
Lyo (32 h)	LOQ	0.24 ± 0.1	0.17 ± 0.02	39.6 ± 4.0	30.1 ± 1.0
Lyo (48 h)	LOQ	0.19 ± 0.17	0.2 ± 0.01	60.1 ± 2.7	29.1 ± 0.6
Fresh petals	LOQ	LOQ	0.02 ± 0	5.0 ± 0.7	3.3 ± 0.4
Max	0.45	2.04	0.52	60.1	30.1
Min	LOQ	LOQ	0.02	5.01	3.3
Mean	0.15	0.41	0.27	21.6	20.3
